# Blood Urea Nitrogen (BUN) is independently associated with mortality in critically ill patients admitted to ICU

**DOI:** 10.1371/journal.pone.0191697

**Published:** 2018-01-25

**Authors:** Okan Arihan, Bernhard Wernly, Michael Lichtenauer, Marcus Franz, Bjoern Kabisch, Johanna Muessig, Maryna Masyuk, Alexander Lauten, Paul Christian Schulze, Uta C. Hoppe, Malte Kelm, Christian Jung

**Affiliations:** 1 Department of Physiology, Van Yuzuncu Yil University Faculty of Medicine, Van, Turkey; 2 Division of Cardiology, Pulmonology, and Vascular Medicine, Medical Faculty, University Duesseldorf, Duesseldorf, Germany; 3 Clinic of Internal Medicine II, Department of Cardiology, Paracelsus Medical University of Salzburg, Salzburg, Austria; 4 Clinic of Internal Medicine I, Department of Cardiology, Jena University Hospital, Jena, Germany; 5 Department of Cardiology, Charité–Universitaetsmedizin Berlin, Berlin, Germany; 6 Deutsches Zentrum für Herz-Kreislauf-Forschung (DZHK), Standort Berlin, Berlin, Germany; UMR UT3 CNRS, FRANCE

## Abstract

**Purpose:**

Blood urea nitrogen (BUN) was reported to be associated with mortality in heart failure patients. We aimed to evaluate admission BUN concentration in a heterogeneous critically ill patient collective admitted to an intensive care unit (ICU) for prognostic relevance.

**Methods:**

A total of 4176 medical patients (67±13 years) admitted to a German ICU between 2004 and 2009 were included. Follow-up of patients was performed retrospectively between May 2013 and November 2013. Association of admission BUN and both intra-hospital and long-term mortality were investigated by Cox regression. An optimal cut-off was calculated by means of the Youden-Index.

**Results:**

Patients with higher admission BUN concentration were older, clinically sicker and had more pronounced laboratory signs of multi-organ failure including kidney failure. Admission BUN was associated with adverse long-term mortality (HR 1.013; 95%CI 1.012–1.014; p<0.001). An optimal cut-off was calculated at 28 mg/dL which was associated with adverse outcome even after correction for APACHE2 (HR 1.89; 95%CI 1.59–2.26; p<0.001), SAPS2 (HR 1.85; 95%CI 1.55–2.21; p<0.001) and several parameters including creatinine in an integrative model (HR 3.34; 95%CI 2.89–3.86; p<0.001). We matched 614 patients with admission BUN >28 mg/dL to case-controls ≤ 28mg/dL corrected for APACHE2 scores: BUN above 28 mg/dL remained associated with adverse outcome in a paired analysis with the difference being 5.85% (95%CI 1.23–10.47%; p = 0.02).

**Conclusions:**

High BUN concentration at admission was robustly associated with adverse outcome in critically ill patients admitted to an ICU, even after correction for co-founders including renal failure. BUN might constitute an independent, easily available and important parameter for risk stratification in the critically ill.

## Introduction

Parameters eligible for risk stratification in critically ill patients are of utmost interest to optimize patients`outcome. Elaborated scoring tools such as APACHE and SAPS scores are available and well validated–still, their complexity and reliance on volatile parameters such as heart rate limits their use especially in initial patients`risk stratification [1, 2].

Markers of renal function are known to be associated with mortality (not only) in cardiovascular disorders [3, 4]. Parameters used for evaluating patients’ renal function and fluid status are glomerular filtration rate (GFR), BUN (Blood Urea Nitrogen) and Creatinine (Cr) [5]. Although Cr is known to be predictive for adverse outcome, BUN was shown to have even additive value in heart failure patients [6, 7]. Further, BUN was shown to be an important marker for metabolic diseases and nutritional status of patients. BUN was evaluated for association with mortality in various settings: Liu et al [8] showed that BUN level predicts hospital mortality in acute aortic dissection patients. In another study, it was shown that elevated BUN constitutes an independent factor in risk stratification of acute necrotizing pancreatitis patients [9]. In patients suffering from peripheral artery disease, high BUN was associated with critical limb ischemia [10].

Both Cr and BUN are filtered from glomerulus due to their small molecular sizes. However, Cr is scarcely reabsorbed from tubules and excreted from the body with urine. On the other hand, urea is reabsorbed from the tubules and urea cycle also has an important physiological role in glomerular water balance. Therefore, BUN is a marker for both neurohumoral activity and renal function as urea reabsorption is altered not only by renal but also endocrine disturbances [11].

BUN is a key element reflecting the intricate interrelation between nutritional status, protein metabolism and renal situation of the patient. BUN might therefore constitute a useful parameter for predicting outcome in critically ill patients. In this study, we aimed to evaluate admission BUN level in critically ill patients admitted to an intensive care unit (ICU) for prognostic relevance.

## Methods

### Study subjects

4176 patients that were treated at the ICU of the Jena University Hospital between January 2004 and December 2009 were enrolled in this study. Main admission diagnoses were pneumonia (n = 533), pulmonary embolism (n = 153), acute coronary syndrome (n = 1909), sepsis (n = 544) and heart failure (n = 611). Inclusion criteria were admission to the medical ICU, and a documented BUN at admission. Standard laboratory values, patient’s medical history and clinical data were documented. All laboratory values were obtained from standard in-hospital laboratory. Follow-up of patients was performed retrospectively between May 2013 and November 2013. The primary endpoint of the study was all cause mortality. Mortality data were collected by review of medical records in our COPRA patient data management system (COPRA System GmbH, Berlin, Germany) and/or patient contact. Data on in-hospital mortality was available for 3847 patients and for long-term mortality on 4059 patients. The study has been approved by the local ethics committee of the University Hospital Jena and waived the requirement for written consent. All data were fully anonymized before evaluation. Minimal anonymized data set is made accessible without restrictions.

### Statistical analysis

Normally distributed data points are expressed as mean ± standard error of the mean. Differences between independent groups were calculated using ANOVA. Categorical data are expressed as numbers (percentage). Chi-square test was applied to calculate differences between groups. In-hospital mortality was assessed by both univariate and multivariate logistic regression. Univariate and multivariate Cox regression analysis was used to evaluate and to adjust for cofounding factors for long-term mortality. For the multivariate regression model, cofounders with a p-value <0.10 in the univariate analysis were included, then a backward variable elimination was performed. Elimination criterion was a p-value of more than 0.10. A p-value of <0.05 was considered statistically significant. We report 3 multivariate regression models. For case-control matching we matched patients in the in the BUN > 28mg/dL group vs the ≤ 28 mg/dL group on APACHE2 score at admission. We could match 614 patients based on these criteria. To compare in-hospital mortality between these paired groups we used McNemar’s test.

SPSS version 22.0 (IBM, USA) and MedCalc version 17.4.4 (MedCalc Software, USA) were used for all statistical analyses.

#### Calculation of SAPS2 and APACHE score

Initial Simplified Acute Physiology Score II (SAPS2) and Acute Physiology And Chronic Health Evaluation (APACHE) scores were calculated by the treating physician within 24 hours after admission as reported before.

## Results

### Study population

Baseline characteristics are shown in Table 1. Patients with higher admission BUN were older (70±12 years vs 62±14 years), clinically sicker as expressed by both higher SAPS2 (52±20 points vs 32±16 points; p<0.001) and APACHE2 (27±9 points vs 16±9 points; p<0.001) scores. Patients with higher BUN had more pronounced laboratory signs of multi-organ failure including liver (ALAT: 3.6±10.6 μmol/l*s vs 1.2±3.9 μmol/l*s; p<0.001; ASAT 7.2±24.9 μmol/l*s vs 3.0±6.6 μmol/l*s; p<0.001).

**Table 1:** Laboratory and clinical baseline characteristics. 4176 medical patients were split in three cohorts according to their BUN concentration at admission, comparison of means by ANOVA.

	BUN 0–20 mg/dL		BUN 20–40 mg/dL		BUN >40mg/dL		overall cohort		
	n = 1820		n = 1296		n = 1060		n = 4176		
	mean	SEM	mean	SEM	mean	SEM	mean	SEM	p-value
age	62.2	13.8	70.1	11.7	69.9	12.2	66.6	13.4	<0.001
SAPS2 (pts)	31.7	16.0	44.3	19.3	51.8	18.8	42.2	19.9	<0.001
APACHE2 (pts)	16.2	8.6	22.2	8.9	26.6	8.5	21.5	9.6	<0.001
lactate (mmol/L)	2.0	3.4	2.8	3.1	3.3	4.0	2.6	3.5	<0.001
PCT (mmol/L)	5.6	15.6	13.4	38.5	14.5	33.8	12.0	32.5	0.001
glucose (mmol/L)	9.0	3.4	10.6	4.1	10.9	3.9	10.0	3.9	<0.001
haemoglobine (mmol/L)	8.2	3.6	7.6	1.1	7.3	4.6	7.8	3.4	<0.001
ASAT (μmol/l*s)	3.0	6.6	5.0	18.1	7.2	24.9	4.9	17.5	<0.001
ALAT (μmol/l*s)	1.2	3.9	2.4	7.4	3.6	10.6	2.3	7.6	<0.001
max_Gamma_GT_μmol	1.4	2.5	1.7	2.2	2.1	2.3	1.7	2.4	<0.001
max_Bilirubin_ges_μmol	16.7	19.4	19.3	26.7	31.2	56.6	21.3	35.5	<0.001
leucocytes (G/L)	10.8	5.1	12.5	9.0	14.2	15.8	12.2	10.1	<0.001
BUN (mg/dL)	13.8	3.7	27.9	5.6	72.6	32.4	33.1	29.1	<0.001
creatinine (mg/dL)	84.0	26.6	135.9	81.0	307.4	215.7	156.8	149.5	<0.001
sodium (mmol/L)	139.5	4.3	140.7	5.1	140.6	7.1	140.1	5.4	<0.001
potassium (mmol/L)	4.0	0.4	4.1	0.6	4.3	0.7	4.1	0.6	<0.001

### Survival data

**Fig 1:**
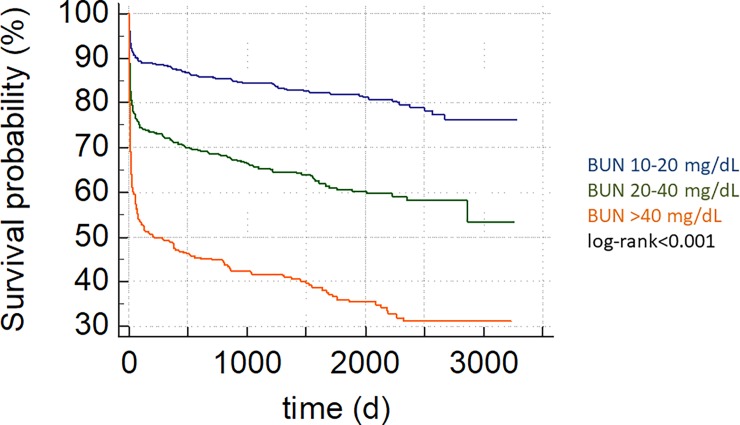
Higher admission BUN levels are associated with adverse long-term outcome, depicted as Kaplan-Meier curve, group comparison by log-rank test, p-value <0.001.

**Fig 2:**
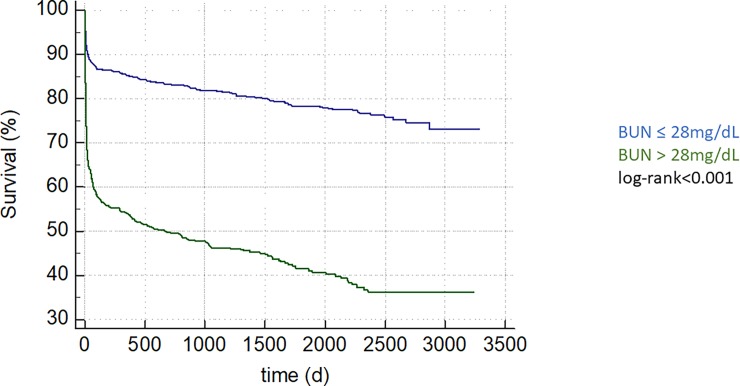
An admission BUN concentration above 28mg/dL, the optimal cut-off calculated by Youden Index, is associated with long term mortality, depicted as Kaplan-Meier curve, group comparison by log-rank test, p-value <0.001.

Patients with BUN above 40 mg/dL evidenced adverse in-hospital outcome compared to the 10–20 mg/dL group (HR 6.05; 95%CI 4.63–7.90; p<0.001; Table 2, Fig 1). Admission BUN was associated with adverse long-term mortality (HR 1.013; 95%CI 1.012–1.014; p<0.001) regardless of admission diagnosis (Table 3). We performed ROC-analysis for BUN (AUC 0.67 95%CI 0.65–0.69) and compared it to SAPS2 (AUC 0.76 95%CI 0.74–0.78; p<0.0001 vs BUN) and APACHE2 (AUC 0.74 95%CI 0.72–0.76; p<0.001 vs BUN). Further, an optimal admission BUN cut-off was calculated at 28 mg/dL. This cut-off was associated with both in-hospital (HR 4.16; 95%CI 3.39–5.10; p<0.001) as well as with long-term mortality (HR 3.74; 95%CI 3.29–4.25; p<0.001, Fig 2).

**Table 2:** Higher admission BUN levels are associated with adverse in-hospital outcome. Hazard ratios (HR) were obtained by logistic regression analysis.

BUN	HR	95%CI	p-value
10–20 mg/dL	1.00		
20–40 mg/dL	2.32	1.75–3.08	<0.001
>40 mg/dL	6.05	4.63–7.90	<0.001
>28 mg/dL	4.16	3.39–5.10	<0.001

**Table 3:** Admission BUN concentration is associated with long-term mortality regardless of admission diagnosis [pneumonia (n = 533), pulmonary embolism (n = 153), acute coronary syndrome (ACS; n = 1909), sepsis (n = 544) and heart failure (AHF, n = 611)]. Hazard ratios (HR) were obtained by Cox regression analysis.

admissio diagnosis	HR	95%CI	p-value
overall cohort	1.013	1.012–1.014	<0.001
sepsis	1.005	1.002–1.008	<0.001
AHF	1.008	1.004–1.012	<0.001
pneumonia	1.008	1.004–1.011	<0.001
pulmonary embolism	1.034	1.024–1.044	<0.001
ACS	1.029	1.026–1.033	<0.001

An admission BUN >28 mg/dL was associated with long-term mortality regardless of admission diagnosis (Table 4). The association of admission BUN >28 mg/dL with long-term mortality remained even after correction for APACHE2 (HR 1.89; 95%CI 1.59–2.26; p<0.001), SAPS2 (HR 1.85; 95%CI 1.55–2.21; p<0.001) and several parameters including creatinine in an integrative model (HR 3.34; 95%CI 2.89–3.86; p<0.001; Table 5).

**Table 4:** An admission BUN concentration above 28mg/dL is associated with long-term mortality regardless of admission diagnosis [pneumonia (n = 533), pulmonary embolism (n = 153), acute coronary syndrome (ACS; n = 1909), sepsis (n = 544) and heart failure (AHF, n = 611)]. Hazard ratios (HR) were obtained by Cox regression analysis.

admission diagnosis	HR	95%CI	p-value
overall cohort	3.74	3.29–4.25	<0.001
sepsis	1.77	1.33–2.36	<0.001
AHF	1.76	1.33–2.32	<0.001
pneumonia	1.59	1.21–2.10	0.002
pulmonary embolism	5.8	3.27–10.26	<0.001
ACS	5.41	4.25–6.90	<0.001

**Table 5:** An admission BUN concentration above 28mg/dL is associated with mortality after correction for several cofounders in a multivariate analysis. Hazard ratios (HR) were obtained by Cox regression analysis, for the multivariate regression models, a backward variable elimination was performed. Elimination criterion was a p-value of more than 0.10.

multivariate model 1						
	univariate			multivariate		
	HR	95%CI	p-value	HR	95%CI	p-value
BUN >28 mg/dL	3.74	3.29–4.25	<0.001	1.89	1.59–2.26	<0.001
APACHE2	1.07	1.07–1.08	<0.001	1.07	1.06–1.08	<0.001
multivariate model 2						
	univariate			multivariate		
	HR	95%CI	p-value	HR	95%CI	p-value
BUN >28 mg/dL	3.74	3.29–4.25	<0.001	1.85	1.55–2.21	<0.001
SAPS2	1.04	1.03–1.04	<0.001	1.03	1.03–1.04	<0.001
multivariate model 3						
	univariate			multivariate		
	HR	95%CI	p-value	HR	95%CI	p-value
BUN >28 mg/dl	3.74	3.29–4.25	<0.001	3.34	2.89–3.86	<0.001
creatinine (mg/dL)	1.001	1.001–1.001	<0.001	1.02	0.98–1.05	0.44
age (y)	1.02	1.02–1.03	<0.001	1.02	1.01–1.02	<0.001
sex (m/w)	0.97	0.86–1.08	0.55	0.9	0.79–1.03	0.9
lactate (mmol/L)	1.06	1.06–1.07	<0.001	1.07	1.06–1.07	<0.001

This was true for the association of admission BUN > 28 mg/dL with intra-ICU mortality, which remained associated with mortality after correction for SAPS2 (HR 1.70 95%CI 1.27–1.27; p<0.001) as well as APACHE2 (HR 1.78 95%CI 1.34–2.38; p<0.001).

### Matched-control analysis

**Fig 3:**
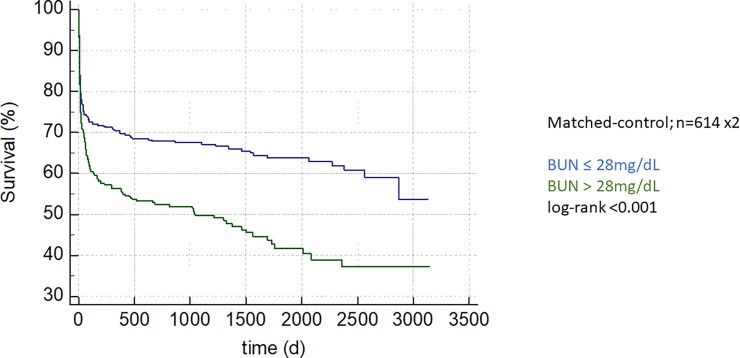
An admission BUN concentration above 28mg/dL, the optimal cut-off calculated by Youden Index, is associated with long term mortality in a matched-control analysis of 614 patients matched on APACHE2 scores, depicted as Kaplan-Meier curve, group comparison by log-rank test, p-value <0.001.

We matched 614 patients with admission BUN >28 mg/dL to case-controls evidencing admission BUN < 28mg/dL corrected for APACHE2 scores: The BUN > 28mg/dL group evidenced adverse intra-hospital outcome in a paired analysis with the difference being 5.85% (95%CI 1.23–10.47%; p = 0.02). Regarding long-term mortality, again, patients in the > 28mg/dL group evidenced adverse outcome (Fig 3; log-rank <0.001).

## Discussion

Identifying patients at high risk for death as soon as possible is of utmost interest in critically ill patients. In our study BUN was independently associated with mortality in critically ill patients admitted to a medical ICU. This association existed regardless of admission diagnosis and remained statistically significant even after correction for relevant cofounders in several multivariate analyses.

This association of BUN with mortality is in accordance to literature: Regardless of admission diagnosis such as acute pulmonary embolism [12], acute pancreatitis [13], acute decompensated heart failure [14], upper gastrointestinal bleeding [15, 16] or ventilation failure which requires mechanical aid [17] high admission BUN levels were reported to be associated adverse outcome. Our study supports this notion in critically ill medical patients.

ROC analysis in this study revealed an optimal admission BUN cut-off of 28 mg/dL which is relatively low, especially in critically ill patients. But, similarly Filippatos et al [7] also found highest 60-day mortality in patients suffering from heart failure in the highest BUN quartile (BUN>40 mg/dL). Even subtle BUN changes comparing patients evidencing BUN concentration above 25 mg/dL against BUN levels between 20 to 25 mg/dL were reported to be predictive for mortality [18]. Therefore, our results support existing literature in this aspect and add substantial knowledge in the heterogeneous patient collective admitted to an ICU.

BUN is known to increase with age and BUN has to be interpreted with caution in elderly [18]. Still in our study cohort, even after correction for age in a multivariate analysis BUN remained predictive for mortality.

In a clinical setting, BUN is mostly interpreted and evaluated with special emphasis on renal failure and fluid balance. On the other hand, BUN is known to be influenced by neurohumoral activation in heart failure and by protein metabolism. In this study, we could show in a multivariate analysis, that BUN was robustly associated with mortality even after correction for renal function–indicating an additive role of BUN to Cr. Patients admitted to an ICU constitute a heterogeneous patient collective. Still, in our study BUN was associated with adverse outcome regardless of admission diagnosis, whereas a mechanistic analysis why and how BUN adds information useful for risk stratification is beyond the scope of this study.

There are sophisticated tools available for risk stratification such as APACHE 2 and SAPS 2 scores which are widely used for risk stratification in critically ill patients. Still, these scores a relatively complex, take some time to calculate and take both volatile parameters—such as heart rate—and parameters which are not always available upon admission—such as concomitant diseases—into account: Therefore, there certainly is a need for a simple tool for quick initial risk stratification. BUN concentration is easy to obtain and a straightforward cut-off such 28 mg/dL can easily be interpreted. This value is also similar to the cut-off used in the tried and tested CURB-65 score for pneumonia severity and might be combined with simple clinical assessments such as initial blood pressure or jugular venous pressure to quickly and easily assess critically ill patient when admitted to an ICU or even an emergency department [19]. At least in our study cohort, high BUN concentration upon admission remained associated with adverse outcome even after correction for both SAPS2 and APACHE2 in a multivariate analysis. As it was even predictive for mortality in a matched-control analysis, we think BUN might constitute a simple and powerful tool robustly associated with mortality for early risk stratification in critically ill patients. In future studies, our BUN cut-off of 28 mg/dL should be prospectively evaluated in the critically ill. One could speculate about combining two simple and cheap modalities such as e.g. BUN concentration and clinical evaluation of fluid status e.g. using jugular vein pressure.

### Conclusion

High BUN concentrations upon ICU admission correctly identify patients who are clinically sicker and evidence more pronounced laboratory signs of multi-organ failure. Admission BUN concentrations were robustly associated with both, intra-hospital and long-term mortality in our cohort study. As this remained true both after correction for several relevant co-founders in a multivariate analysis as well is in matched-control analysis, we think that BUN might constitute an independent risk factor, with additive value to renal failure, possibly indicating neurohumoral activation and disturbed protein metabolism. BUN is easy to measure and might therefore be a potent tool for risk stratification of critically ill patients.

## Supporting information

S1 File 171220_minumum_data_BUN_BW.xlsx contains all relevant information. (XLSX)
